# Outcomes of the Modified Cabrol Technique in Aortic Root Replacement: Early and Midterm Experience

**DOI:** 10.1093/icvts/ivaf235

**Published:** 2025-09-27

**Authors:** Nayeem Nasher, Joshua Chen, Purab Kothari, Colin King, Jacqueline McGee, Vishal Shah, Saarah Khairi, Konstadinos Plestis

**Affiliations:** Department of Cardiac Surgery, Thomas Jefferson University Hospital, Philadelphia, PA 19107, United States; Sidney Kimmel Medical College, Thomas Jefferson University, Philadelphia, PA 19107, United States; Sidney Kimmel Medical College, Thomas Jefferson University, Philadelphia, PA 19107, United States; Department of Cardiac Surgery, Thomas Jefferson University Hospital, Philadelphia, PA 19107, United States; Department of Cardiac Surgery, Thomas Jefferson University Hospital, Philadelphia, PA 19107, United States; Department of Cardiac Surgery, Thomas Jefferson University Hospital, Philadelphia, PA 19107, United States; Department of Cardiac Surgery, Thomas Jefferson University Hospital, Philadelphia, PA 19107, United States; Department of Cardiac Surgery, Thomas Jefferson University Hospital, Philadelphia, PA 19107, United States

**Keywords:** aortic root replacement, modified Cabrol technique, coronary interposition graft, anticoagulation

## Abstract

**Objectives:**

The modified Cabrol technique has been associated with excellent graft patency. However, prior studies were limited to patients largely on long-term anticoagulation. We sought to analyse outcomes in patients primarily managed without mechanical prostheses.

**Methods:**

We retrospectively analysed all patients who underwent aortic root replacement by a single surgeon from 2014 to 2024. Patients who underwent reimplantation of one or both coronary ostia using the modified Cabrol technique with separate interposition grafts were identified. Baseline characteristics and postoperative outcomes were reported. Predictors of mortality were analysed using Cox proportional hazards, and overall survival was reported using Kaplan-Meier analysis.

**Results:**

We identified 91 patients who underwent the modified Cabrol technique. The median age was 62 [IQR, 52-71] years, and 91.2% (83/91) were male. Patients presented urgently or emergently in 38.5% (35/91) of cases, for acute dissection in 23.1% (21/91) of cases, and for endocarditis in 15.4% (14/91). Patients required redo sternotomy in 50% of cases. A mechanical composite valve graft was used in only 7.7% (7/91) of patients. The incidence of long-term myocardial infarction was 4.4% (4/91). Survival at 1 and 5 years was 93% and 89%, respectively. There was no significant association with the utilization of Cabrol graft and long-term mortality (hazard ratio 1.74, 95% CI 0.76-4.01, *P* = .219).

**Conclusions:**

Patients undergoing the modified Cabrol technique had an acceptable risk of mortality in short- and midterm follow-up. The modified Cabrol technique is a valuable tool in an aortic surgeon’s arsenal and should be used selectively.

## Graphical abstract

**Figure:**
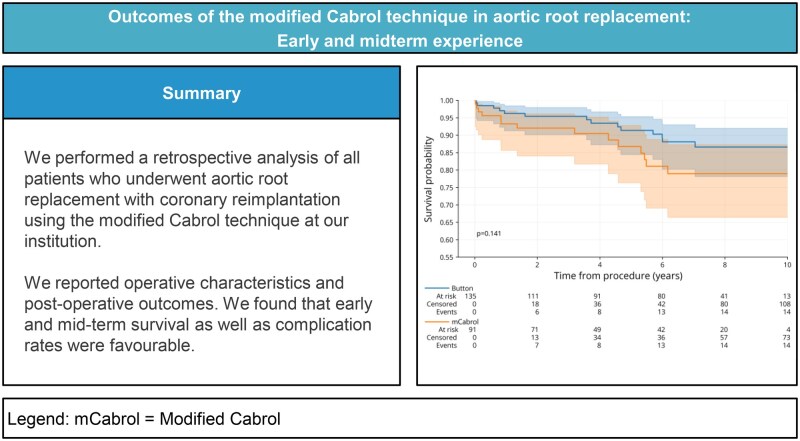

## INTRODUCTION

The optimal technique for management of the coronary ostia in aortic root replacement (ARR) remains a challenge, particularly in patients requiring reoperation, those with dissections, and those with anatomical abnormalities.^1^ The Carrel button technique has become the standard approach in straightforward cases due to its reliability and simplicity, but it remains a challenge in select scenarios due to the risk of kinking or occlusion. Inadequate length and difficult mobilization can lead to tension on the anastomosis and potentially catastrophic dehiscence.^2^ The original Cabrol technique, which utilizes a single-tube graft in a “mustache” configuration by which the 2 ends are anastomosed to the right and left coronary ostia in an end-to-end fashion, was designed to facilitate a tension-free anastomosis of the coronary arteries, offering an alternative to the direct aortocoronary anastomosis in the Bentall procedure, especially in cases where there is considerable challenge in mobilizing the coronaries.^3^ However, the length of the graft makes it prone to kinking, and computational studies have shown that the side-to-side nature of the anastomosis results in spiralling fluid dynamics and non-laminar flow, making it prone to thrombosis.^4^ As such, the technique has been reserved for complex ARR, such as in redo operations, where coronary mobilization can be limited.

The original Cabrol procedure has since undergone several modifications, including the utilization of short interposition grafts anastomosed end-to-end to each coronary ostium, with each graft separately anastomosed to the aortic root graft in an end-to-side configuration. Presently, few studies have evaluated the long-term outcomes of the modified Cabrol technique (mCabrol).^5–8^ Furthermore, most series analysing long-term patency are limited to mostly mechanical composite valve grafts (mCVG) requiring anticoagulation. With the utilization of valve-sparing techniques and biological composite valve grafts (bCVG), additional data on Cabrol graft patency in patients without anticoagulation are increasingly relevant. We describe the largest comparative study in which the modified Cabrol technique was utilized for coronary reconstruction in ARR, primarily with tissue valves.

## METHODS

### Study design

A prospectively maintained aortic database was queried for all patients who underwent ARR between June 2014 and June 2024. ARR was performed with an mCVG, bCVG, or valve-sparing root replacement by a single surgeon. Patients who underwent selective sinus replacement or the Florida sleeve procedure were excluded. Patients who underwent coronary artery reattachment using either unilateral or bilateral Dacron interposition grafts were considered to have undergone the mCabrol procedure. Approval for the study was granted by the Institutional Review Board on September 13, 2023 (iRISID-2023-2417), and informed consent was waived due to the retrospective nature of the study.

**Figure 1.**
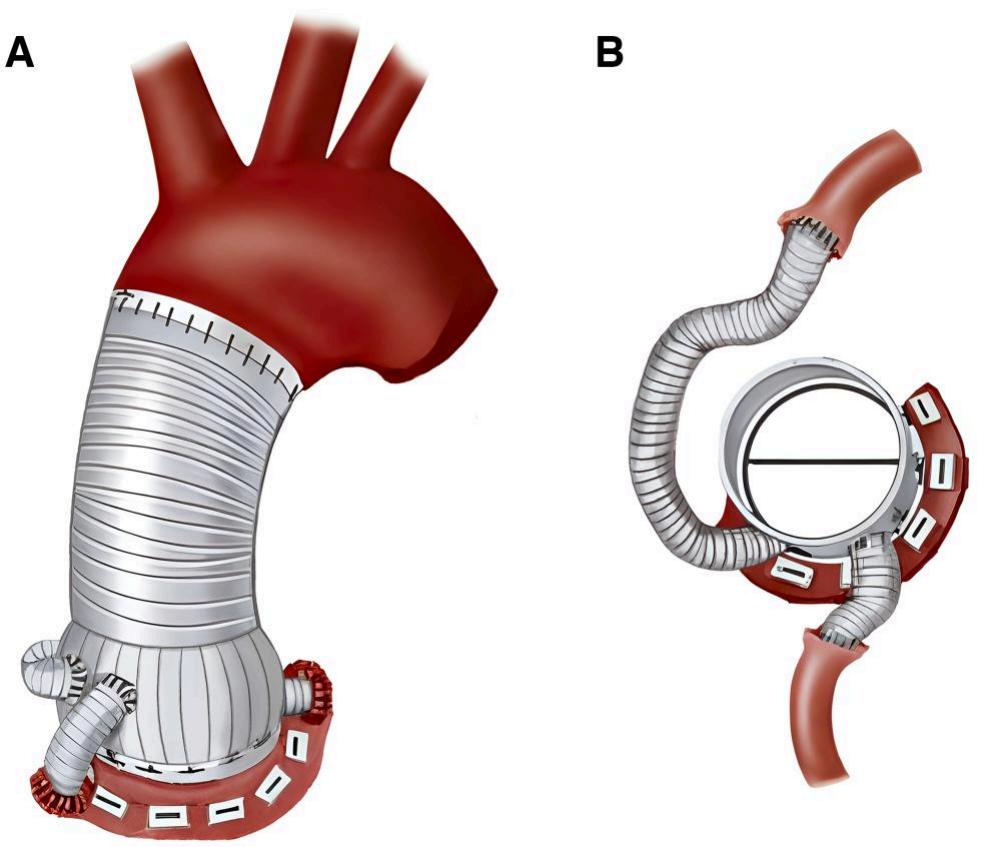
Schematic Depicting the Modified Cabrol Coronary Artery Reattachment Technique. (A) Anterior oblique view. (B) Craniocaudal view

The mCabrol technique was employed when the coronary ostia are low to the annulus, anomalous in origin, widely displaced, immobile secondary to adhesions/dissection, or deemed too fragile to mobilize. Either a 6 mm, 8 mm, or 10 mm Dacron graft was chosen to best match the size of the coronary ostia. For the proximal anastomoses, the right main graft was placed anteriorly, and the left main graft was placed in the right anterolateral position and courses posterior to the root graft (**Figure 1**). The height of the anastomoses is roughly 1 cm above the neo-annulus and is kept at the same level in cases of bilateral reattachment. All patients were routinely discharged on 90 days of aspirin 81 mg daily postoperatively, and long-term anticoagulation was only utilized for mCVGs.

Patients were followed annually in our aortic wellness clinic and received postoperative CT scans annually to assess for complications, as well as graft patency. Baseline and intraoperative characteristics, along with postoperative outcomes, were reported.

### Statistical analysis

Baseline patient characteristics and postoperative outcomes were analysed accordingly. A complete case analysis was performed, in which patients with missing outcome measures and key variables were excluded from our analysis. Normality was assessed with the Kolmogorov-Smirnov test, and data were determined to be non-normally distributed. Therefore, categorical variables were reported as frequencies with percentages, and continuous variables were reported as medians with IQRs. Kaplan-Meier survival analysis was performed. Multivariable Cox regression analysis was performed to assess long-term survival and was conducted using clinically significant preoperative variables on univariate analysis. Collinearity was assessed using the variance inflation factor. The results of the analysis were reported as hazard ratios (HR) with 95% CIs. *P*-values <.05 were considered significant. Statistical analyses were done using R software, version 4.4.1 (R Foundation for Statistical Computing, Vienna, Austria).

## RESULTS

### Baseline characteristics

We identified 91 patients who underwent the modified Cabrol technique during ARR. The median age was 62 [IQR, 52-71] years, and 91.2% (83/91) of patients were male. Medical comorbidities were notable for diabetes in 6.6% (6/91), hypertension in 79.1% (72/91), congestive heart failure in 17.6% (16/91), and coronary artery disease in 14.3% (13/91). Surgical history was notable for prior cardiac surgery in 49.5% (45/91) of patients who required redo sternotomy. At the time of presentation, 38.5% (35/91) underwent an urgent or emergent operation, with acute type A aortic dissection (ATAD) in 23.1% (21/91) and endocarditis in 15.4% (14/91). Baseline characteristics and operative indications are further outlined in **Table 1**.

**Table 1.** Baseline Preoperative Characteristics

Characteristic	Cabrol (*N* = 91)
Age (years), median [IQR]	62 [52-71]
Male	83 (91.2%)
Diabetes	6 (6.6%)
Hypertension	72 (79.1%)
Hyperlipidemia	42 (46.2%)
CAD	13 (14.3%)
Prior/current smoker	35 (38.5%)
Congestive heart failure	16 (17.6%)
COPD	6 (6.6%)
Peripheral artery disease	7 (7.7%)
Renal insufficiency	4 (4.4%)
Prior stroke	9 (9.9%)
Bicuspid aortic valve	15 (16.5%)
Marfan syndrome	1 (1.1%)
BMI (kg/m^2^), median [IQR]	27.9 [24.9-31.6]
NYHA dyspnoea	
Class I	61 (67.0%)
Class II	12 (13.2%)
Class III	12 (13.2%)
Class IV	6 (6.6%)
Aortic insufficiency	
None	32 (35.2%)
Mild	16 (17.6%)
Moderate	19 (20.9%)
Severe	24 (26.4%)
Symptoms at presentation	
Chest pain	23 (25.3%)
Shortness of breath	20 (22.0%)
Asymptomatic	38 (41.8%)
Prior cardiac surgery	45 (49.5%)
Prior proximal aortic surgery	15 (16.5%)
Preoperative LVEF (%), median [IQR]	60 [55-65]
Indication for surgery	
Degenerative aneurysm	31 (34.1%)
Acute dissection	21 (23.1%)
Chronic dissection	9 (9.9%)
Endocarditis	14 (15.4%)
Urgent or emergent	35 (38.5%)

Abbreviations: BMI, body mass index; CAD, coronary artery disease; COPD, chronic obstructive pulmonary disease; LVEF, left ventricular ejection fraction; NYHA, New York Heart Association.

### Operative characteristics

Bilateral Cabrol interposition grafts were utilized in 37.4% (34/91) of cases, right main-only interposition graft in 46.2% (42/91), and left main-only interposition graft in 16.5% (15/91) of cases. For the right coronary, an 8 mm interposition graft was used in 55.3% (42/76) and a 6 mm graft in 39.5% (30/76) of the cases. For the left coronary, an 8 mm interposition graft was used in 73.5% (36/49), and a 6 mm graft in 12.2% (6/49) of the cases. An mCVG was used in 7.7% (7/91) of patients, a bCVG in 65.9% (60/91), and valve-sparing root replacement was performed in 16.5% (15/91). A concomitant aortic arch replacement was performed in 18.7% (17/91) of cases, and deep hypothermic circulatory arrest (DHCA) was utilized in 31.9%. The median cardiopulmonary bypass time was 241 minutes [IQR, 204-281], and the median cross-clamp time was 184 minutes [IQR, 164-231]. Further operative details are noted in **Table 2**.

**Table 2.** Operative Details and Characteristics

Characteristic	Cabrol (*N* = 91)
Bentall	67 (73.6%)
Bioprosthetic	60 (65.9%)
Mechanical	7 (7.7%)
Valve sparing	15 (16.5%)
Xenograft	9 (7.7%)
CPB time (minutes), median [IQR]	241 [204-281]
Cross clamp time (minutes), median [IQR]	184 [164-231]
Circulatory arrest	31 (34.1%)
Circulatory arrest time (minutes), median [IQR]	21 [6-28]
DHCA	29 (31.9%)
Coronary reimplantation	
Right and left main interposition graft	34 (37.4%)
Right main only interposition graft	42 (46.2%)
Left main only interposition graft	15 (16.5%)
Interposition graft size, *n*/*N* (%)	
Right 10 mm	4/76 (5.3%)
Right 8 mm	42/76 (55.3%)
Right 6 mm	30/76 (39.5%)
Left 10 mm	7/49 (14.3%)
Left 8 mm	36/49 (73.5%)
Left 6 mm	6/49 (12.2%)
Concomitant procedures	
Arch repair	17 (18.7%)
CABG	10 (11.0%)
MVR	8 (8.8%)
Blood products (units), median [IQR]	
PRBC	2 [2-5]
FFP	2 [0-4]
Cryoprecipitate	1 [1-4]
Platelets	2 [1-3]

Abbreviations: CABG, coronary artery bypass graft; CPB, cardiopulmonary bypass; DHCA, deep hypothermic circulatory arrest; FFP, fresh frozen plasma; LVEF, left ventricular ejection fraction; PRBC, packed red blood cells.

### Early outcomes

Postoperatively, the median left ventricular ejection fraction was 60% [IQR, 55-60]. Prolonged ventilation greater than 48 hours occurred in 34.1% (31/91), open chest management in 27.5% (25/91), and renal dysfunction in 5.5% (5/91) of cases. The median ICU length of stay was 5 [IQR, 3-10] days, and the median hospital length of stay was 11 [IQR, 6-19] days. In-hospital mortality was 3.3% (3/91). Further postoperative outcomes are outlined in **Table 3**.

**Table 3.** Postoperative Outcomes and Long-Term Complications

Characteristic	Cabrol (*N* = 91)
Post-operative LVEF (%), median [IQR]	60 [55-60]
ICU length of stay (days), median [IQR]	5 [3-10]
Ventilator time (days), median [IQR]	1 [1-3]
Hospital length of stay (days), median [IQR]	11 [6-19]
Postoperative complications	
Prolonged ventilation	31 (34.1%)
Chest left open	25 (27.5%)
Pneumonia	13 (14.3%)
Pleural effusion	4 (4.4%)
Re-intubation	6 (6.6%)
Renal dysfunction	5 (5.5%)
Required hemodialysis	4 (4.4%)
Bleeding	7 (7.7%)
Stroke	2 (2.2%)
Low cardiac output	2 (2.2%)
Postoperative MI^a^	0 (0%)
Late MI	4 (4.4%)
Coronary pseudoaneurysm	1 (1.1%)
Reoperation proximal aorta	3 (3.3%)
In-hospital mortality	3 (3.3%)
30-day mortality	2 (2.2%)
1-year mortality	6 (6.6%)
5-year mortality	10 (11.0%)
Overall mortality	14 (15.4%)
Follow-up time (years), median [IQR]	3.1 [0.9-6.5]
Latest CT-scan (years), median [IQR]	2.3 [0.8-5.0]

Abbreviations: ICU, intensive care unit; LVEF, left ventricular ejection fraction; MI, myocardial infarction.

a MI occurring within 30 days of surgery.

### Midterm outcomes

**Figure 2.**
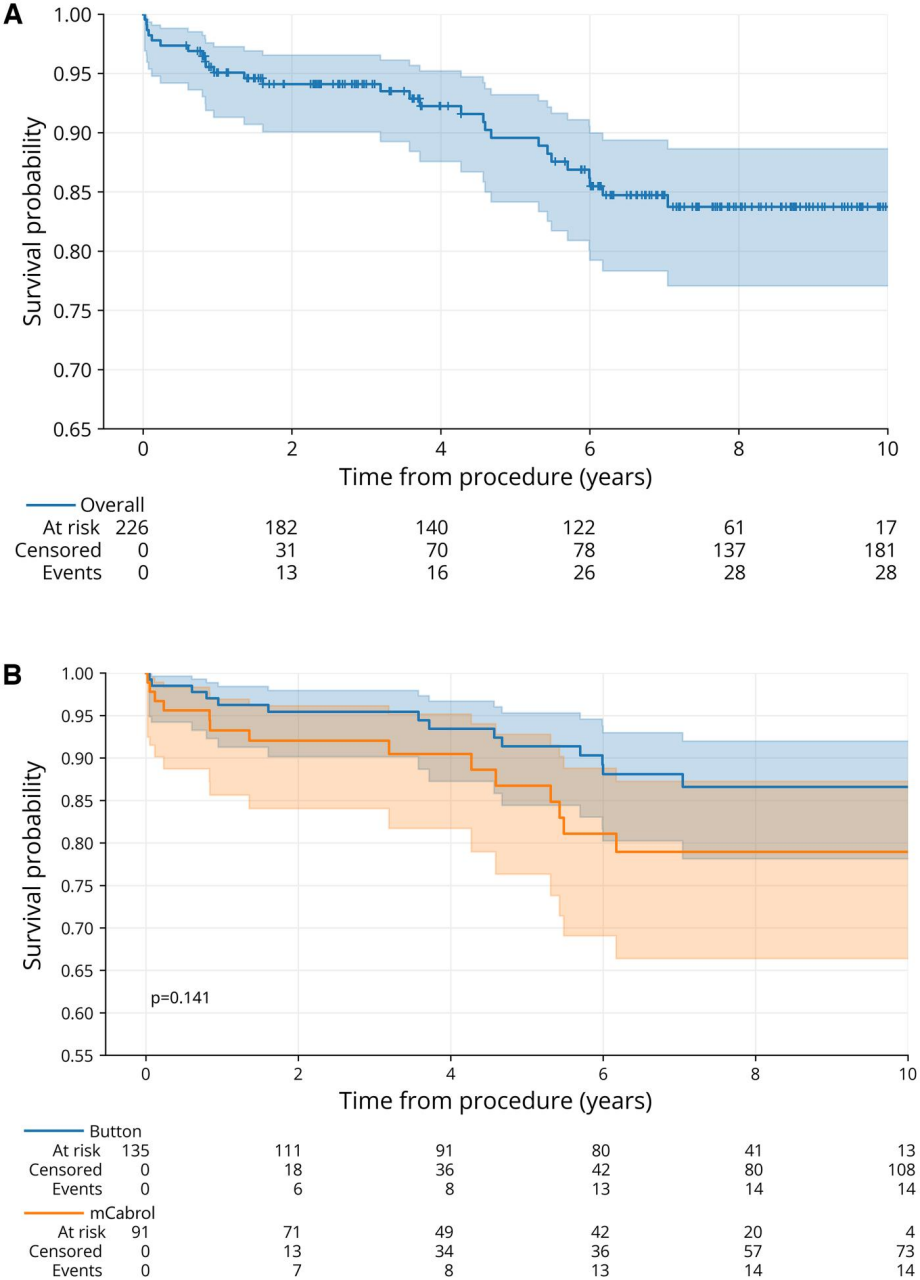
Kaplan-Meier Survival Analysis with 95% CIs. (A) Overall survival probability. (B) Survival probability according to procedure type

Postoperative CT scans were available in 87% of all patients following hospital discharge. The latest radiographic follow-up was obtained at a median time of 2.3 years postoperatively. Proximal aortic reoperation occurred in 3.3% (3/91). Four cases of late myocardial infarction occurred after hospital discharge. See **Table 4** for further case details. Freedom from mortality at 1 and 5 years was 93% and 89%, respectively. There was no difference in overall survival in patients undergoing ARR with or without mCabrol grafts (log-rank, *P* = .141) (**Figure 2**). Multivariable Cox regression analysis for follow-up mortality showed no significant association between utilization of Cabrol grafts and survival (HR 1.74, 95% CI: 0.76-4.01). Further results of the analysis are noted in **Table 5**.

**Table 4.** Characteristics of Patients with Late MI in the Modified Cabrol Group

Patient No.	1	2	3	4
Age	78	67	45	52
Relevant history	VF ablation, PPM, TIA, SAVR	Sick sinus syndrome, PPM, TIA	None	CKD, VSRR
Indication for surgery	AVR dehiscence	Aortic root dilatation	Aortic dissection	Root abscess
Operation	Redo ARR with CVG, MVR	ARR with CVG	VSRR, ascending aorta, and arch replacement	Redo ARR with CVG, ascending aorta replacement
Coronary findings	Friable ostia, adhesions	Displaced right coronary	Dissected right coronary	Friable ostia
Valve type	Bioprosthetic	Bioprosthetic	Native	Bioprosthetic
Cabrol type	Right main—6 mm Left main—8 mm	Right main only—8 mm	Right main only—6 mm	Right and left main—8 mm
Antiplatelet regimen^a^	Aspirin 81 mg daily	Aspirin 81 mg daily	None	None
Coronary complication	Left main graft occlusion	Right main pseudoaneurysm with graft occlusion	Right main graft occlusion	Right main graft occlusion
Time from surgery to event	51 months	21 months	4 months	40 months
Outcome	Death	Failed balloon angioplasty—extensive collateralization	RCA stent	RCA stent

Abbreviations: ARR, aortic root replacement; AVR, aortic valve replacement; CKD, chronic kidney disease; CVG, composite valve graft; MI, myocardial infarction; MVR, mitral valve replacement; PPM, permanent pacemaker; RCA, right coronary artery; SAVR, surgical aortic valve replacement; TIA, transient ischaemic attack; VF, ventricular fibrillation; VSRR, valve-sparing root replacement.

a At the time of the event.

**Table 5.** Multivariable Cox Regression Analysis for Long-Term Mortality

Variable	Hazard ratio	95% CI	*P*-value
Cabrol graft	1.74	0.76-4.01	.219
Symptoms at presentation	0.42	0.12-1.45	.182
NYHA dyspnoea class IV	4.18	1.19-14.70	.026
Prior stroke	3.91	1.22-12.55	.018
Acute dissection	4.24	1.09-18.19	.012
Urgent/emergent	0.90	0.16-5.03	.489

Abbreviation: NYHA, New York Heart Association.

## DISCUSSION

Our study demonstrated that the modified Cabrol technique can be safely implemented in complex ARR with careful consideration for long-term patency. Early reports have suggested several advantages of the original Cabrol technique over the button technique, such as circumventing the need to dissect the coronary buttons and providing easier access to coronary suture lines in cases of bleeding.^9^ Given the relative immobility of the right coronary artery, the use of a Cabrol graft helps minimize kinking, torsion, and dehiscence in complex aortic surgery. It is also particularly useful when addressing the left main button, as the posterior location of the left coronary ostium relative to the graft can limit visualization and complicate hemostasis. Nevertheless, the Cabrol technique has mostly fallen out of favour due to concerns over long-term graft patency, as several studies have demonstrated its inferiority.^9–11^

The modified Cabrol technique sought to address its shortcomings by simplifying the technique to incorporate 2 individual grafts anastomosed to the root graft in an end-to-side configuration. These modifications commonly utilize 2 short interposition grafts, which would ideally allow for a more physiologic flow compared to the mustache technique. We elected to place the proximal anastomosis of the left main graft in the right anterolateral position of the root graft to maintain direct visualization in case of bleeding. Additionally, the height of the anastomosis for both grafts is as close to the neo-annulus as anatomically permissible to minimize the length of the graft to avoid kinking. Additionally, when choosing the size of the Cabrol graft, we try to match the size of the graft to the size of the coronary to preserve physiological flow dynamics as much as possible.

Only a handful of studies have assessed the long-term durability of the modified Cabrol technique. Most have shown favourable outcomes regarding graft patency, though mortality rates vary significantly according to the patient population being treated. Hirasawa et al reported no coronary complications with a survival rate of 94% at 5 years.^5^ Maureira et al described only one instance of coronary pseudoaneurysm with no loss of graft patency and an overall survival rate of 74% at 10 years.^6^ Both studies report the use of an mCVG in 100% of cases, which may facilitate Cabrol graft patency owing to the need for lifelong anticoagulation postoperatively.^12^

In a series of 40 patients, Ziganshin et al demonstrated 100% graft patency rates with a mean radiographic follow-up time of 3.3 years.^7^ They described the use of mechanical prostheses in 63% of their patients and reported an overall survival rate of 73% at 6 years. In the most recent series preceding ours, Tanaka et al reported outcomes of the modified Cabrol technique in a particularly high-risk group, in which 74% of patients underwent redo sternotomy at the time of presentation and 40% had a prior type A aortic dissection repair.^8^ In their series of 84 patients, at a median radiographic follow-up time of approximately 3 years, they reported coronary complications requiring intervention in 4% of cases, but no instance of Cabrol graft occlusion. Of note, operative mortality was 15% in their cohort compared to 3.3% in ours, despite both studies reporting outcomes for all-comers.

In our series, we reported the use of a mechanical valve in only 8% of patients. Thus, to the best of our knowledge, our study describes the largest series of patients who underwent the modified Cabrol procedure without the need for long-term anticoagulation postoperatively for a mechanical valve. This may be due to several differences in our cohort compared to prior studies, namely, the utilization of valve-sparing techniques whenever possible in younger patients and an older median age of our cohort, in which a bioprosthetic valve may be more appropriate. Consequently, we report a loss of graft patency in 4 patients, though several factors must be considered. Two patients developed right interposition graft occlusions at 4 and 40 months, respectively, while exercising, and were brought to the catheterization laboratory and had stents placed without complication. A third patient had a right interposition graft pseudoaneurysm and occlusion at 21 months. A balloon angioplasty was attempted without success; however, the patient had already developed extensive collateralization. The last patient was a 78-year-old male who had occlusion of the left Cabrol graft at 51 months and ultimately passed away. Secondary patency was achieved in 2 of the 4 cases with coronary stenting, and the grafts remain patent to date. Otherwise, there were no instances of luminal narrowing. It is possible that the excellent patency rates in previous studies can be attributed to the need for anticoagulation, as some of the authors have suggested.^7^ However, given the high mortality rates in prior studies (up to 15%) and the low number of events of loss of patency in ours, it is difficult to identify specific risk factors (dissection, endocarditis, graft length, post-operative anticoagulation) associated with long-term loss of patency. To date, there is no clear consensus on the use of antiplatelet or anticoagulation for coronary interposition grafts.

In our study population, patients presented with a considerable operative risk, with a need for redo sternotomy in 50%, along with a higher acuity of presentation for indications such as ATAD in 23%, and root abscess or endocarditis in 15% of cases. Additionally, the complexity of operation was reflected in the need for DHCA in 32% of cases, and the prevalence of arch involvement necessitating a complex arch repair. While many of these factors may lend to the higher rate of postoperative complications as well as a prolonged ICU and hospital length of stay, long-term survival outcomes were favourable.

### Limitations

This study is subject to several limitations, including those inherent to retrospective review, such as documentation errors, incomplete follow-up, and the lack of randomization with the potential for confounders. Particularly, there may be an underreporting of adverse events in patients lost to follow-up or who have transferred care to an outside institution where medical records cannot be easily retrieved. A major limitation of the study is that all procedures were performed by a single surgeon, which limits the external validity of our study. We acknowledge the presence of selection bias, as our study population was limited to patients referred to a single provider at a metropolitan tertiary care centre. These factors limit the generalizability of our study.

## CONCLUSIONS

The modified Cabrol technique is a safe and valuable tool in the armamentarium of an aortic surgeon, particularly in the application of challenging coronary mobilization in the setting of high-risk, redo operations.

## Data Availability

The data underlying this article will be shared on reasonable request to the corresponding author.
